# The Impact of COVID-19 Pandemic and Lockdown on Alcohol Consumption: A Perspective From Hair Analysis

**DOI:** 10.3389/fpsyt.2021.632519

**Published:** 2021-04-06

**Authors:** Eugenio Alladio, Lia Visintin, Tonia Lombardo, Roberto Testi, Alberto Salomone, Marco Vincenti

**Affiliations:** ^1^Dipartimento di Chimica, Università degli Studi di Torino, Torino, Italy; ^2^Centro Regionale Antidoping e di Tossicologia “A. Bertinaria”, Torino, Italy

**Keywords:** ethyl glucuronide, hair test, alcohol, COVID-19, addiction

## Abstract

**Introduction and Aims:** The increase in stress levels, social confinement, and addiction's physical consequences play an essential role in the proliferation of drug abuse. In this context, the Covid-19 pandemic produced remarkable effects on those individuals prone to addictions, especially to alcohol. Alcohol is linked to multiple dangerous conditions such as social issues, severe medical conditions, and road accidents. The determination of ethylglucuronide (EtG) in hair is frequently performed to test and monitor chronic excessive alcohol intake conditions, as it allows differentiation among low-risk/moderate drinkers, and excessive/chronic drinkers. Our study aimed to explore hair EtG levels in a controlled population to assess the impact of Covid-19 lockdown on alcohol intake along March-May 2020.

**Materials and Methods:** EtG levels were measured in all hair samples collected in the months following April 2020 to evaluate the behaviors related to alcohol intake along with the time frame from March to May 2020. The measured concentration distributions for each month were compared with those reported in the same month during the previous 4 years (2016–2019). The dataset was built to highlight possible differences between genders, and the different categories of alcohol consumption, separately.

**Results:** The samples collected from April to August 2020 (500 < *N* <1,100 per month) showed an increase in the percentage of subjects classified as abstinent/low-risk drinkers (from 60 up to 79%) and a decrease of subjects classified as moderate and chronic drinkers (−12 and −7%, respectively) when compared to the previous 4 years. A decrease in the overall mean value of EtG in the period April–June 2020 was observed, while the EtG levels of both June and July 2020 provided an increasing trend for chronic/excessive consumers (+27 and +19% for June and July 2020, respectively). A peculiar rise in the EtG levels of moderate and chronic/excessive female consumers was observed along April–June 2020, too.

**Discussion and Conclusions:** Behavioral and social studies generally report a decrease in alcohol consumption during the Covid-19 lockdown. However, people already suffering from drug or alcohol addictions before Covid-19 pandemic seemingly enhance their harmful behavior. Our data from April to August 2020 are consistent with both suppositions. Our observations confirm once again the utility of EtG to investigate the patterns of alcohol consumption in the population.

## Introduction and Aims

During 2020, the World Health Organization (WHO) and the European section of WHO (1) have published several studies and report (2–5) about alcohol consumption and alcohol addiction dealing with fundamental issues in preventing risks and alcohol-related harm. In the last report published by WHO in 2020 (3), it is stated that alcohol is the primary cause of deterioration in health, disability, and premature death in Europe, which ranks first globally in terms of alcohol consumption. The impact of alcohol is mainly recorded on people of working and productive age. Thus, alcohol is a factor that might hinder economic development and represent an additional financial burden for the society, with consequences for health systems and criminal justice that largely outweigh the benefits of income tax on alcoholic products. Alcohol is not only a significant risk factor for non-communicable diseases (such as cancer and heart disease), but it also contributes to the spread of infectious diseases, and considerable increase in mental health problems, road accidents, injuries, violent accidents, and crimes (1). For these reasons, the National Alcohol Observatory for Italy (ONA) repeatedly expressed concern about the COVID-19 pandemic (https://www.epicentro.iss.it/alcol/epidemiologia-monitoraggio-2020) (6) and its impact on alcohol consumption. The 2020 ONA report states that the growth in the consumption of pure alcohol *per capita* between 2018 and 2019 continues to increase and has reached a level of 7 L/year (7). An increase in the number of consumers between meals, consumers at risk, and binge drinkers (i.e., those who consume large quantities of alcohol in limited periods, for example during the weekend) has been observed, too. 14.2% of men and 6.1% of women reported that they routinely consumed excess alcoholic beverages. In Italy, 6.2 M of male consumers and 2.5 M of female consumers revealed that they did not comply with the public health indications regarding the frequency, the quantity of alcohol, and the alcohol consumption of alcoholic beverages, so that currently a total of 8.7 M individuals have to be considered “at risk” in Italy.

During the Sars-CoV-2 pandemic, WHO Europe has published the document “Frequently asked questions about alcohol and Covid-19” (8) concerning the relationship between the effects of alcohol and the virus spread. The WHO emphasizes that alcohol addiction during an emergency is dangerous from two points of view. First, there is an increased likelihood of being infected by the virus and adverse health outcomes since alcohol compromises the body's immune system. Severe alcohol abuse is actually a risk factor for pneumonia and other lung infections such as the development of acute respiratory distress syndrome (ARDS), which is one of the main complications of Covid-19. Secondly, rising levels of stress, isolation, withdrawal symptoms (i.e., tremors, nausea, and cravings), combined with more difficult access to services and support groups may increase people's risks with alcohol dependence. Several studies have demonstrated the correlation between exposition to catastrophic or stressful events and addiction or increase in alcohol consumption (9–11), even if other studies do not confirm these results (12). Adams et al. (9) investigated the relationship between alcohol consumption and mental health in the context of terrorist attacks. The results showed that binge drinking is related to post-traumatic stress disorder (PTSD) syndromes, while alcohol dependence is related to PTSD, depression, somatization, anxiety, and low quality of life. The same results were confirmed by Lebeaut et al. (11) in a study carried out on firefighters. Boscarino et al. (10) studied alcohol abuse disorder in the period following the 11th September 2001 terroristic attack in New York, highlighting a remarkable aptitude for binge drinking in the immediate aftermath, as well as a long-term increase in alcohol consumption and addiction. However, these findings have been disproved by other studies [for instance, North et al. (12)] that highlighted a 22% increase in PTSD in the population that survived flood disasters, often in comorbidity with depression, but they did not detect any increase or development of dependence on alcohol or other substances. Therefore, the stress arising after a traumatic event is not likely to be the only factor influencing the state of alcohol abuse and substance addiction. For instance, Wu et al. (13) identified the high degree of exposure to a virus and isolation as significant and contributing factors to alcohol abuse and substance addiction when evaluating the data collected during the SARS epidemic emergency over 3 years. Columb et al. (14) hypothesized that another influencing factor is the existence of previous states of dependence by observing an increase in the number of people turning to the help-desks for addictions during the Sars-CoV-2 (Covid-19) emergency. Consequently, the isolation and lack of distractions created by social distancing, possibly in conjunction with increased stress, anxiety, and boredom, may lead to the development of alcohol abuse disorders or relapse into pre-existing alcohol addictions (14). A further study by the University of Padua (15) showed that 66.0% of the people answering to a diet modification questionnaire concerning the quarantine period increased the consumption of “comfort food”. 42.7% of them declared that this increase was due to an increase in the anxiety level. Furthermore, it was reported that alcohol consumption decreased by 36.8% and increased by 10.1% of the tested population. It is essential to highlight that 78% of the study's statistical population was under 35 years old, and alcohol consumption preferentially occurs in the form of social drinking for the selected age range.

The present study aims to assess the impact of the Covid-19 emergency on alcohol intake and addiction for the population of North-Western Italy by monitoring ethyl glucuronide (EtG) concentration in hair as a direct biomarker of ethanol consumption. The determination of EtG in the keratin matrices has gained an increasing appreciation since it achieves the highest combination of sensitivity and specificity in the discrimination among alcohol consumers with different drinking habits (16–21). Thus, the determination of EtG in hair is nowadays widely accepted for testing and monitoring chronic excessive alcohol intake, and it is currently employed in different areas of forensic and clinical toxicology, including workplace testing, firearms, driving license re-granting, and post-mortem investigation (17, 22, 23). The data used in the present study have been collected at the Anti-doping and Toxicology Center “A. Bertinaria” of Orbassano (Torino, Italy) (24) from 2016. The hair EtG analytical results arose from samples collected from subjects who underwent medical examination within driving re-granting protocols, alcohol abuse rehabilitation programs, or workplace testing.

## Materials and Methods

### Datasets

This study evaluates the Covid-19 pandemic and lockdown impacts on the population's alcohol intake in the time frame from January 2020 to May 2020 by measuring the EtG level in hair samples collected with the appropriate time-shift. The lockdown protocol started in Italy on March 8th 2020, and it finished at the end of May 2020. Considering that hair grows ~1 cm/month and, commonly, the proximal head hair segment with a length of 3 cm is analyzed (19, 25–27), only hair samples collected from April 2020 to August 2020 were selected for this study. On average, the effect of a change in the amount of alcohol consumed is observed with a delay of about 2 months.

The hair samples were analyzed at the Anti-doping and Toxicology Center “A. Bertinaria” of Orbassano (Torino, Italy) (https://www.antidoping.piemonte.it/cms/) and refer to a population resident in Northern-Western Italy. More in detail, the selected population includes subjects aiming to regain their driving license temporarily suspended for administrative/legal sanctions, individuals under continuous monitoring due to their ongoing or past alcohol-dependence conditions, and professional workers undergoing workplace testing. No exclusion criteria were applied in the study. Although the Center's database contains reports about the EtG levels in hair that date back to 2011, we decided to assess the impact of the Covid-19 emergency on alcohol consumption by building a dataset containing the EtG values of the last 5 years only, from 2016 to 2020, because the hair sample pre-treatment procedure was modified in the analytical protocol during the Autumn 2015. As a matter of fact, the pulverization of the keratin matrix using a ball mill in place of manual cutting produced an average 38% increase of the detected EtG level (28), as a consequence of an improved extraction yield. The fundamental methodological details of hair analysis are available in published studies (28, 29). To remove the methodological change bias factor from the data, the results before 2016 were not used. The EtG values measured on the samples collected during each month were averaged (April to August, 2020) and compared with the corresponding monthly-averaged values reported in the previous 4 years (i.e., 2016–2019). This approach based on the comparison of data collected in the same month of different years was adopted because the occurrence of a seasonal variation of the average EtG values was observed in a previous study (30).

The collected dataset was split into sub-groups depending on the gender of the tested individuals and their classification into three categories, namely abstinent/low-risk drinkers, social/moderate drinkers, and chronic/excessive drinkers (21) following the Society of Hair Testing (SoHT) guidelines about the use of EtG in hair for supporting the assessment of abstinence and chronic alcohol consumption. The classification is based on the following cut-off values:

- Abstinent/low-risk drinkers (labeled as *Abs*): EtG <10 pg/mg;
- Social/moderate drinkers (labeled as *SDr*): 10 pg/mg ≤ EtG <30 pg/mg;
- Chronic/excessive drinkers (labeled as *Chr*): EtG ≥ 30 pg/mg.

The monthly-averaged EtG values (April–June) for the different years (2016–2020) were compared for the different categories of gender and alcohol consumption, to highlight the effects of the Covid-19 emergency. Since the original database reported a “lower than 10 pg/mg” output for *Abs*-labeled samples, for statistical purposes, a random value between 1 and 9 pg/mg was arbitrarily assigned to them. A comprehensive table reporting the numbers of samples involved in the study is available in the Supplementary Table 1 with details about the number of male and female individuals in the different alcohol consumption categories.

### Statistics and Data Interpretation

The first phase of data interpretation evaluated the absolute values, percentage frequencies, and percentage differences for the various categories of alcohol consumption and gender. In the second step, the variations of EtG levels for each month over the years was studied by plotting their EtG mean values, together with 95% confidence intervals and the number of individuals involved. Lastly, analysis of variance (ANOVA) (31) and Kruskal–Wallis test (32) were used to determine whether the differences found in the previous phases had statistical significance or, conversely, had to be ascribed to random statistical fluctuation in the collected data.

#### Statistical Tests

In ANOVA (31) and Kruskal–Wallis tests, the continuous dependent variable was the concentration of ethyl glucuronate in the keratin matrix, while the investigated factors included the individuals gender and the time of sample collection (months or years). Consequently, the levels are male/female for the gender factor and the years (2016, 2017, 2018, 2019, and 2020) or the months (April, May, June, July, and August) for the time. With this analysis, it is possible to compare different distributions or groups of data and, according to their variance, confirm the existence of dissimilar distributions, trends, or anomalous results. Assumptions involving the probability distribution of the data, their independence and absence or outliers were tested before performing ANOVA and Kruskal–Wallis tests, as follows: (i) normality was tested using QQ-plots, (ii) the homogeneity of the variance within the groups (i.e., homoscedasticity) was tested via Bartlett's test (32, 33).

Once ANOVA identified a significant statistical difference, additional evaluations involving Tukey's HSD (honestly significant difference) (32, 33) tests were performed to determine which group significantly differed from the others [thus performing a multiple comparison procedure (MCP)]. Finally, the results obtained after applying ANOVA were confirmed using the non-parametric Kruskal–Wallis test, since the available data contained many outliers for chronic/excessive drinkers (*Chr*), corresponding to very high levels of EtG (observed in both genders). The results obtained by Tukey's HSD test were verified also by the Wilcoxon–Mann–Whitney rank-sum (non-parametric) test (32).

### Software

Data processing was carried out using R software (version 4.0.2) (34) and R Studio (version 1.3.959) (35). The following packages were used for various representations and statistical analysis: ggplot2 (36), gplots (37), and dplyr (38).

## Results

### Data Structure and Summary

**Figure 1:**
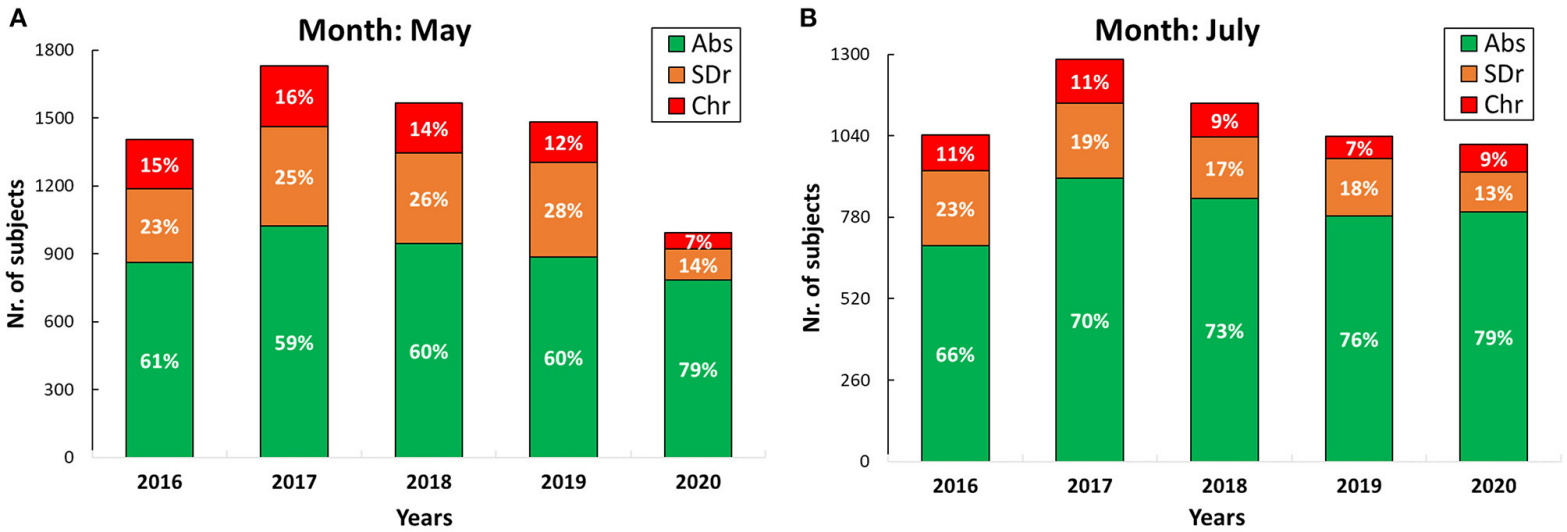
Stacked barplots showing the number and the relative percentages of *Abs* (green), *SDr* (orange), and *Chr* (red) individuals in May **(A)** and July **(B)** 2016–2020.

The total number of samples, the absolute and relative percentage frequencies were calculated for each year and month by considering the different genders and categories of alcohol consumption. Figure 1 shows the number of analyzed hair samples for May (Figure 1A) and July (Figure 1B) 2016–2020. The stacked barplot reports the counts and the relative percentage frequencies of subjects belonging to the three categories [i.e., abstinent/low-risk drinkers (*Abs*), social/moderate drinkers (*SDr*), and chronic/excessive drinkers (*Chr*)]. The number of May 2020 samples (nr. = 992) is significantly lower (~ −36%) than in the same month for the years 2016–2019 (nr. = ~1,546, on average). The months of April, June, and August 2020 showed the same decreasing trend (−53, −18, and −21%, respectively), while July 2020 provided a total number of specimens quite close to the past 4 years (−9%). All the stacked barplots are available in Supplementary Figure 1. Figure 1 also reports the same data in terms of relative percentages for the various classes of alcohol consumers. May 2020 (Figure 1A) shows a distinct increase of the *Abs* subjects (+19%) with respect to the average percentage observed in 2016–2019 (i.e., 79 vs. 60%). Accordingly, lower percentages of *SDr* (−12%) and *Chr* (−7%) individuals are observed. Similar trends are evident from April and June data. In July 2020 (Figure 1B), a slightly higher percentage of *Abs* (and a lower percentage of *SDr*) is still observed with respect to the previous years, while the percentage of *Chr* individuals is approximately the same. In August 2020, the percentage distribution turned back similar to the one observed in the previous 4 years.

Women represent only a small percentage (11%) of the overall dataset; approximately the same percentage was recorded for the entire period 2016–2020. The number of women providing EtG values higher than 10 pg/mg is relatively low (23% of the women, against 41% for the men), as well as the number of chronic/excessive drinkers (7% of the women, against 15% for the men). Further evaluations were made to evaluate potential bias in the sampling of the subjects under evaluation. The individuals were divided into the following three categories: (i) DRL: those seeking driver license reinstatement, (ii) TAA: those tracked for alcohol abuse, and (iii) WT: those seeking workplace testing. The frequencies and percentages of the three types of visitors were calculated for the year 2020 and then monthly-compared with 2016–2019. The results in terms of pie charts and chi-squared tests are available in the Supplementary Figures 2A–K and Supplementary Table 2.

### Evaluation of Mean Values

**Figure 2:**
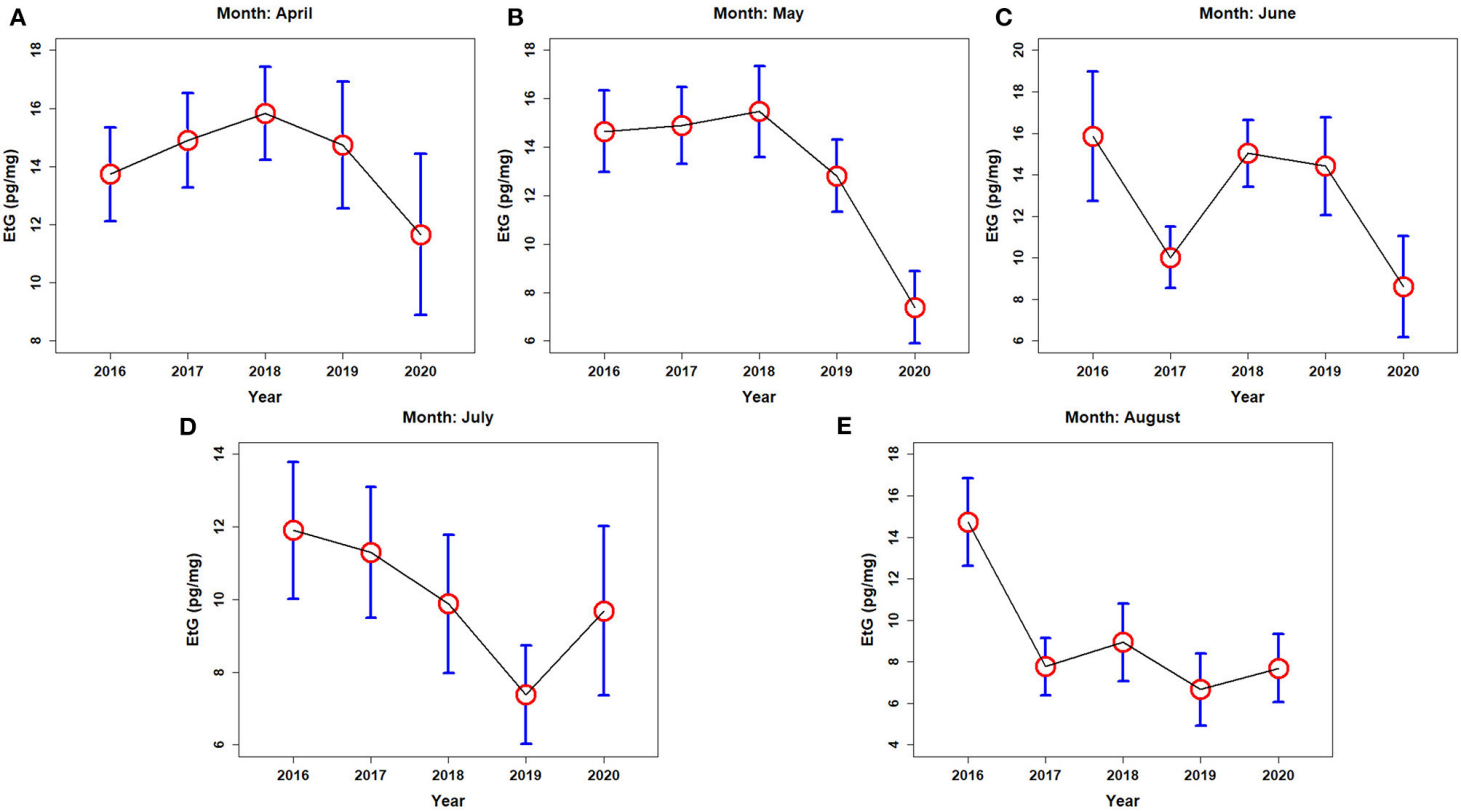
Mean Etg values (red circles) and 95% confidence intervals (blue bars) for all the analyzed hair samples relative to the months of **(A)** April, **(B)** May, **(C)** June, **(D)** July, and **(E)** August 2016–2020.

**Figure 3:**
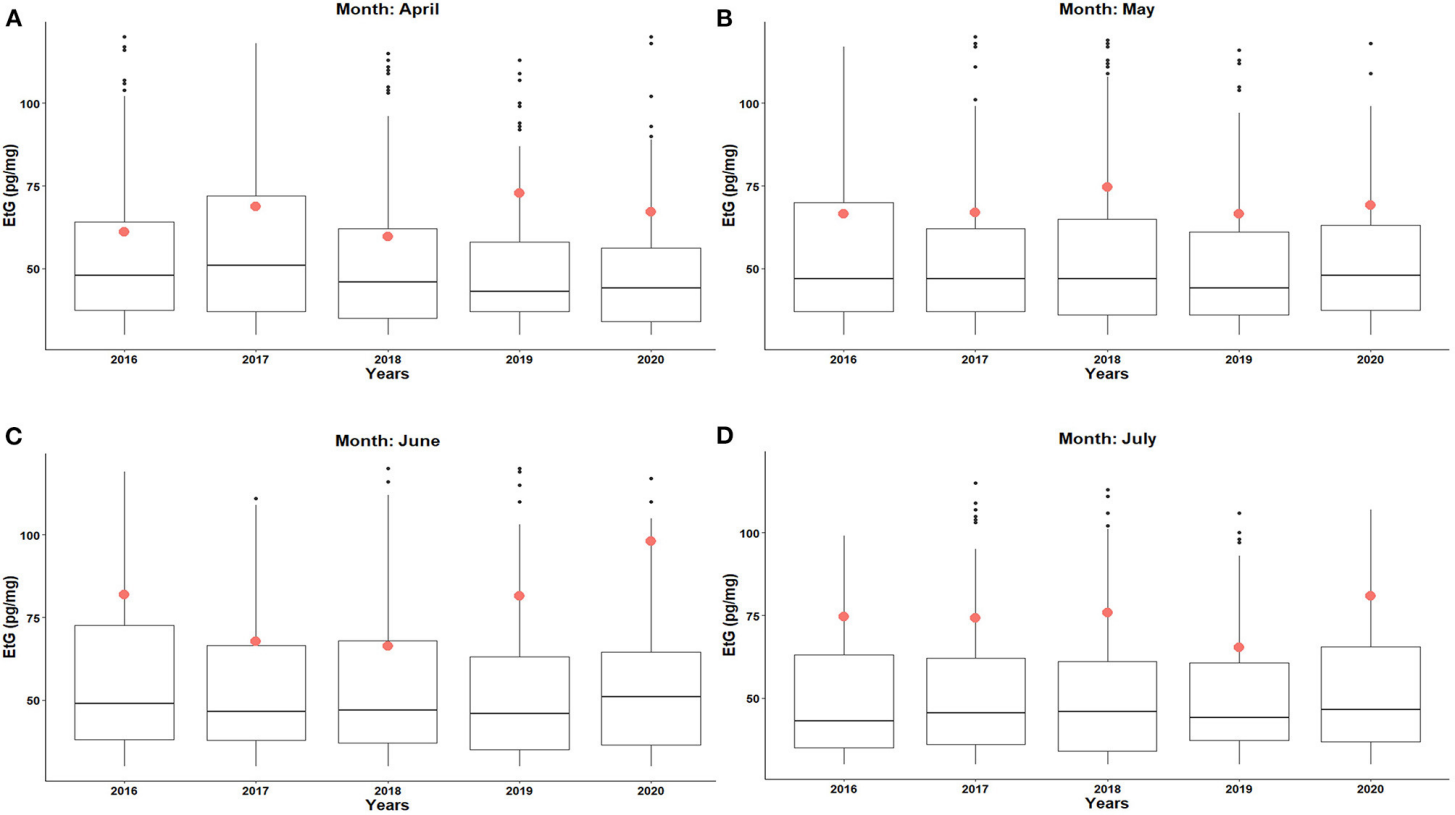
Boxplots and mean Etg values (red circles) for the individuals showing higher EtG levels than 30 pg/mg (i.e., Chr population) concerning the months of **(A)** April, **(B)** May, **(C)** June, and **(D)** July 2016–2020. Black points are the outliers.

Since the number of samples collected from women represents a small fraction of the overall dataset, no differences were made in terms of gender when plotting the mean values of EtG for the different categories of alcohol consumption, together with their 95% confidence intervals (Figures 2, 3). However, a brief focus on the EtG levels of the female population will be brought into at the end of this section. The mean EtG values measured in each month of 2020 were compared with values reported in the same month during the previous 4 years (2016–2019). All the results in terms of total numbers and percentages are reported in Supplementary Tables 1, 2.

Figure 2 shows the mean values for EtG calculated for all the months (April–August) and years (2016–2020). A consistent decrease in the mean EtG values was recorded in the year 2020 for the months of April 2020 (Figure 2A), May (Figure 2B), and June 2020 (Figure 2C). In contrast, the EtG mean value for July 2020 (Figure 2D) and August 2020 (Figure 2E) show comparable results with the previous years.

When the mean EtG values were calculated only from the samples with measurable Etg levels (i.e., higher than 10 pg/mg; the specimens belonging to the *Abs* population were excluded), no significant changes were detected in the year 2020, because the population shift from upper to lower categories of alcohol consumers observed in 2020 gets undetected in single category values (see Supplementary Figure 2). Lastly, Figure 3 depicts the boxplots and the mean EtG values (red circles in Figure 3) calculated from the samples with Etg levels exceeding 30 pg/mg (i.e., the hair specimens belonging to *Chr* populations). In this case, June 2020 (Figure 3B) and July 2020 (Figure 3C) data show a detectable increase in the mean EtG values, while April, May (Figure 3A), and August 2020 provided no change with respect to the previous years.

**Figure 4:**
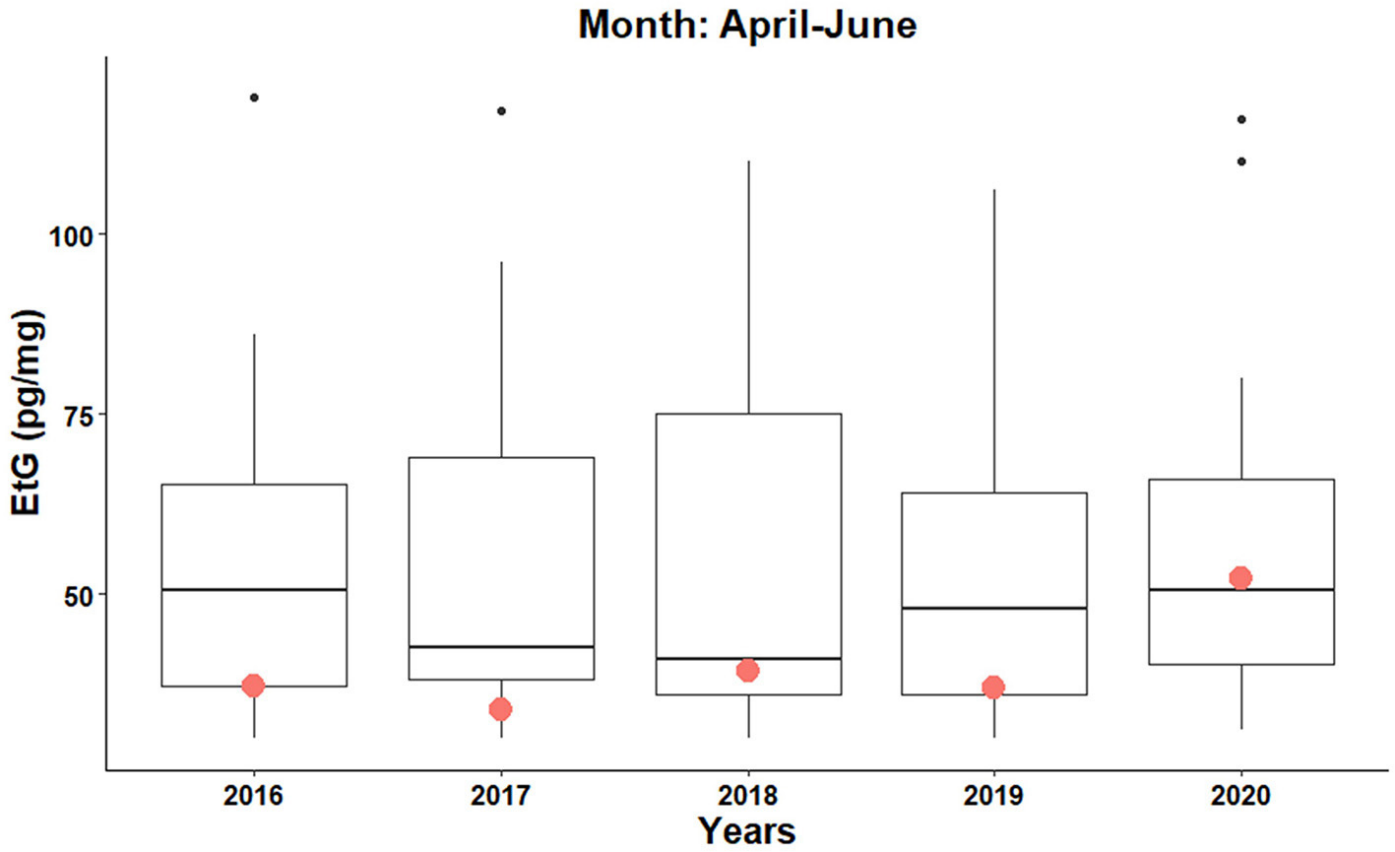
Boxplots and mean Etg values (red circles) for the women showing EtG levels higher than 10 pg/mg in the combined period April–June 2016–2020. Black points are the outliers.

With respect to the women data showing measurable EtG levels (i.e., higher than 10 pg/mg), the combined April–June 2020 period was considered in order to put together a statistically significant population. The results reported in Figure 4 show a peculiar increase of the mean EtG value in the 2020 hair samples with respect to the previous years. In contrast, the mean EtG values recorded in July and August 2020 provided results similar to the 2016–2019 time range (data not shown).

The results plotted in Figure 2 showed wide 95% confidence intervals, especially for the 2020 data, possibly because the Covid-19 emergency reduced the total number of samples collected and simultaneously amplified the inter-individual variability of the results. For these reasons, the use of statistical significance tests turned out necessary to support the observed trends.

### Significance Tests

Parametric tests including ANOVA and Tukey HSD tests were performed to verify the statistical significance of the variations observed in the EtG result distributions during the lockdown period with respect to the corresponding periods of the preceding years.

It was preliminarily checked if the EtG distributions for the different periods April–June 2016–2020 fulfilled the ANOVA application conditions: normality, independence and homogeneous variance. Q–Q plots and Bartlett's test confirmed the subsistence of ANOVA applicability. The presence of scattered outliers in the data distribution, relative to samples with very high EtG values induced us to verify ANOVA and Tukey HSD results with alternative non-parametric approaches (Kruskal–Wallis and Wilcoxon–Mann–Whitney tests). The most important results are listed below:

The EtG distributions relative to May and June 2020 that included all categories of consumers (Figure 2) proved different, with statistically significant *p*-values lower than 0.05 for both the parametric (May: df = 4, *F*_calc_ = 11.76, *p* = 1.61e-09; June: df = 4, *F*_calc_ = 7.74, and *p* = 3.23e-06) and non-parametric tests (May: df_1_ = 992, df_2_ = 6,186, *W* = 2,475,055, *p* = 1.70e-29; June: df_1_ = 1,073, df_2_ = 5,232, *W* = 2,362,794, *p* = 1.65e-22), with respect to the corresponding month of each year along the period 2016–2019, with the only exception of June 2020 vs. June 2017. Mean EtG levels resulted significantly lower in both May and June 2020, but also in April 2020 according to non-parametric tests only.

Considering only the EtG values higher than 10 pg/mg (relative to moderate consumers and chronic/excessive drinkers; Supplementary Figure 2), the differences observed in the global set of data disappears or becomes not statistically significant. Only July 2020 data show increased EtG results with respect to July 2016–2019 corroborated by significant *p*-values (ANOVA: df = 1, *F*_calc_ = 20.42, *p* = 6.39e-06; Wilcoxon–Mann–Whitney: df_1_ = 800, df_2_ = 3,683, *W* = 1,363,260, *p* = 3.13e-06). The loss of significance observed on the upper portion of the data is somehow expected, because the fixed cut-off erases the lower tail of the distributions, leveling off the remaining results.

The limited population involved in the comparison of *Chr* subjects together with the large spread of the experimental EtG values prevent any rational application of rigorous statistical tests. On the whole, the mean EtG levels recorded on both June and July 2020 show an increasing trend in comparison with the mean EtG values recorded in 2016–2019. In detail, June 2020 data correspond to an average EtG value of 94 pg/mg, corresponding to a +27% increase with respect to the average value recorded in June for the years 2016–2019 (74 pg/mg). Similarly, the mean EtG value of July 2020 is 85 pg/mg, showing a +19% difference from July 2016 to 2019 (71 pg/mg). All the percent differences are available in Supplementary Table 3.

Taking into account the moderate and excessive female drinkers (Figure 4), Wilcoxon–Mann–Whitney test provided a significant *p*-value equal to 0.030 (df_1_ = 48, df_2_ = 352, *W* = 10,082) was obtained by comparing the higher EtG levels of April–June 2020 with respect to April–June 2016–2019. In this scenario, the data of April–June 2020 correspond to a mean EtG of 52 pg/mg, showing a + 40% increase with respect to April–June 2016–2019 (37 pg/mg).

## Discussion and Conclusions

Several studies reported significant worsening in the behavior of people already addicted to alcohol, gambling, or drugs after the occurrence of catastrophic or stressful events (2, 9–11, 14). People who suffered from alcohol addiction before the Covid-19 pandemic might relapse into it or aggravate their harmful behavior. On the other hand, Scarmozzino and Visioli (15) described a self-reported decrease in alcohol consumption during Covid-19 lockdown; despite the self-reported results may be underestimated when dealing with alcohol consumption (39), it is plausible that this shift is related to the “social” category of drinkers, whose alcohol consumption commonly takes place outside their households.

Our results are consistent with these evaluations. The comparison of the relative frequencies along 2016–2020 showed a noteworthy increase in the number of abstinent/low-risk drinkers in April (+10.6%), May (+19.0%), and July (+15.2%) 2020. Accordingly, the number of moderate and chronic/excessive drinkers dropped, thus revealing an immediate influence on the drinking habits due to the Covid-19 lockdown of March–May 2020. Moreover, the mean EtG values showed decreasing trends in April, May, and June 2020, indicating a variation in alcohol consumption during the first months of the Covid-19 pandemic. Notably, a change in the drinking habits of the controlled population is expected to show its maximum effect after about 2–3 months, as is actually observed, due to the average rate of hair growth (1 cm/month) and the proximal 3-cm segment undergoing analysis. These results fit with the conclusions available in the literature about low-risk and social categories of drinkers (13, 40), which consume alcohol for its socializing and pleasuring effects.

The interpretation of chronic/excessive consumers showed a reduction in the number of samples and the relative percentage frequencies in 2020 and, simultaneously, a severe intra-variability of the EtG values for this category (Supplementary Figure 3, Figures 3, 4). The chronic/excessive drinkers showed higher mean EtG values in June and July 2020 (+27 and +19%, respectively). This phenomenon is not perceived by evaluating the whole population since the overall dataset contains a large percentage of abstinent and low-risk consumers. Our results corroborate the conclusions reported in several other studies stating that emergencies and trauma may worsen the mid/long-term addiction of high-risk consumers. These people had to face their addiction in a moment of vulnerability caused by anxiety, depression, stress, social isolation, and inability to access any welfare service (12, 14).

The COVID-19 pandemic has been associated with stress-associated and post-infection dermatologic conditions, including hair loss and altered hair growth (41–43). While we can reasonably believe that most of subjects undergoing hair collection were not Covid-positive or been in contact with Covid-positive (otherwise they would have been quarantined), it is impossible to estimate if any bias related to altered hair growth occurred in our population.

Lastly, moderate and chronic/excessive female drinkers showed the highest mean EtG level when the data collected from April to June 2020 are merged. According to our data, they seemed to worsen their drinking habits during the lockdown, while the male excessive drinkers showed the highest mean EtG values in correspondence with the re-opening of bars and restaurants (i.e., June and July 2020). However, it has to be noted that female drinkers represent a small percentage of the study samples.

In conclusion, this study supports the proposition that the Covid-19 emergency and the consequent lockdown condition affected the drinking habits of the different categories of alcohol consumers in several peculiar ways. While the average alcohol intake of social consumers was observed to decrease, on the other hand the consumption from chronic/excessive drinkers showed an alarming increment. Noteworthy, the alcoholic drinks were largely accessible during the lockdown, since supermarkets and liquor stores remained open, and delivering from on-line stores was always possible. On the other hand, bar and restaurants were shut down, thus significantly limiting the opportunities for “social drinking” (44). The cogency of hair EtG as a biomarker for monitoring and retrospective analysis of average alcohol consumption has been proved once again, particularly when large population datasets are available. Future developments of this study will be addressed to the monitoring of the second surge of the Covid-19 infection and particularly concern the long-term influence of the Covid-19 emergency on alcohol addicted patients.

## Data Availability Statement

The raw data supporting the conclusions of this article will be made available by the authors, without undue reservation.

## Ethics Statement

Ethical review and approval was not required for the study on human participants in accordance with the local legislation and institutional requirements. Written informed consent for participation was not required for this study in accordance with the national legislation and the institutional requirements.

## Author Contributions

EA: data elaboration and manuscript draft. LV: experimental and data elaboration. TL: sample analyses. AS: rationale and manuscript draft. MV: manuscript supervision. All authors contributed to the article and approved the submitted version.

## Conflict of Interest

The authors declare that the research was conducted in the absence of any commercial or financial relationships that could be construed as a potential conflict of interest.
