# Supplementary material for: The Impact of COVID-19 Pandemic and Lockdown on Alcohol Consumption: A Perspective From Hair Analysis
Source: Front Psychiatry. 2021 Apr 6;12:632519. doi: 10.3389/fpsyt.2021.632519 (PMC8055823; doi:10.3389/fpsyt.2021.632519)
Supplement: Supplementary file 1 [file Data_Sheet_1.docx]

**Supplementary Material**

| Overall | | | | | | | | | | | | |
| --- | --- | --- | --- | --- | --- | --- | --- | --- | --- | --- | --- | --- |
| Year | Total | Abs | SDr | Chr | ♂ | ♀ | Abs(♂) | SDr(♂) | Chr(♂) | Abs(♀) | SDr(♀) | Chr(♀) |
| 2016 | 5741 | 3642 | 1264 | 835 | 5235 | 506 | 3236 | 1196 | 803 | 406 | 68 | 32 |
| 2017 | 6776 | 4488 | 1454 | 834 | 6119 | 657 | 3941 | 1378 | 800 | 547 | 76 | 34 |
| 2018 | 6402 | 4106 | 1432 | 864 | 5797 | 605 | 3633 | 1345 | 819 | 473 | 87 | 45 |
| 2019 | 6039 | 4029 | 1362 | 648 | 5435 | 604 | 3559 | 1280 | 596 | 470 | 82 | 52 |
| 2020 | 4459 | 3455 | 646 | 358 | 3939 | 520 | 3019 | 604 | 316 | 436 | 42 | 42 |

**Table 1S.** Numbers of samples involved in the study divided by years (2016-2020) and months (April-June). *Abs* stands for abstinent and low-risk drinkers, *SDr* stands for social/moderate drinkers, and *Chr* indicates the chronic/excessive drinkers. Symbols ♂ and ♀ represent the male and female consumers, respectively.

| **2016** | | | | | | | | | | | | |
| --- | --- | --- | --- | --- | --- | --- | --- | --- | --- | --- | --- | --- |
| **Month** | **Total** | **Abs** | **SDr** | **Chr** | ♂ | ♀ | **Abs**(♂) | **SDr**(♂) | **Chr**(♂) | **Abs**(♀) | **SDr**(♀) | **Chr**(♀) |
| **April** | 1107 | 697 | 234 | 176 | 1013 | 94 | 625 | 218 | 170 | 72 | 16 | 6 |
| **May** | 1405 | 861 | 327 | 217 | 1270 | 135 | 753 | 308 | 209 | 108 | 19 | 8 |
| **June** | 1232 | 771 | 283 | 178 | 1125 | 107 | 687 | 266 | 172 | 84 | 17 | 6 |
| **July** | 1042 | 689 | 239 | 114 | 953 | 89 | 617 | 229 | 107 | 72 | 10 | 7 |
| **August** | 955 | 624 | 181 | 150 | 874 | 81 | 554 | 175 | 145 | 70 | 6 | 5 |
|  |  |  |  |  |  |  |  |  |  |  |  |  |
| **2017** | | | | | | | | | | | | |
| **Month** | **Total** | **Abs** | **SDr** | **Chr** | ♂ | ♀ | **Abs**(♂) | **SDr**(♂) | **Chr**(♂) | **Abs**(♀) | **SDr**(♀) | **Chr**(♀) |
| **April** | 1341 | 803 | 338 | 200 | 1218 | 123 | 703 | 322 | 193 | 100 | 16 | 7 |
| **May** | 1732 | 1022 | 441 | 269 | 1555 | 177 | 880 | 417 | 258 | 142 | 24 | 11 |
| **June** | 1348 | 947 | 268 | 133 | 1222 | 126 | 838 | 255 | 129 | 109 | 13 | 4 |
| **July** | 1285 | 904 | 240 | 141 | 114 | 1171 | 807 | 229 | 135 | 97 | 11 | 6 |
| **August** | 1070 | 812 | 167 | 91 | 953 | 117 | 713 | 155 | 85 | 99 | 12 | 6 |
|  |  |  |  |  |  |  |  |  |  |  |  |  |
| **2018** | | | | | | | | | | | | |
| **Month** | **Total** | **Abs** | **SDr** | **Chr** | ♂ | ♀ | **Abs**(♂) | **SDr**(♂) | **Chr**(♂) | **Abs**(♀) | **SDr**(♀) | **Chr**(♀) |
| **April** | 1326 | 721 | 360 | 245 | 1203 | 123 | 635 | 335 | 233 | 86 | 25 | 12 |
| **May** | 1565 | 945 | 401 | 219 | 1407 | 158 | 824 | 376 | 207 | 121 | 25 | 12 |
| **June** | 1337 | 787 | 335 | 215 | 1208 | 129 | 687 | 314 | 207 | 100 | 21 | 8 |
| **July** | 1143 | 839 | 197 | 107 | 1040 | 103 | 750 | 188 | 102 | 89 | 9 | 5 |
| **August** | 1031 | 814 | 139 | 78 | 939 | 92 | 737 | 132 | 70 | 77 | 7 | 8 |
|  |  |  |  |  |  |  |  |  |  |  |  |  |
| **2019** | | | | | | | | | | | | |
| **Month** | **Total** | **Abs** | **SDr** | **Chr** | ♂ | ♀ | **Abs**(♂) | **SDr**(♂) | **Chr**(♂) | **Abs**(♀) | **SDr**(♀) | **Chr**(♀) |
| **April** | 1253 | 764 | 309 | 180 | 1140 | 113 | 676 | 295 | 169 | 88 | 14 | 11 |
| **May** | 1484 | 887 | 417 | 180 | 1342 | 142 | 782 | 396 | 164 | 105 | 21 | 16 |
| **June** | 1315 | 855 | 291 | 169 | 1182 | 133 | 762 | 266 | 154 | 93 | 25 | 15 |
| **July** | 1038 | 784 | 183 | 71 | 929 | 109 | 690 | 173 | 66 | 94 | 10 | 5 |
| **August** | 949 | 739 | 162 | 48 | 842 | 107 | 649 | 150 | 43 | 90 | 12 | 5 |
|  |  |  |  |  |  |  |  |  |  |  |  |  |
| **2020** | | | | | | | | | | | | |
| **Month** | **Total** | **Abs** | **SDr** | **Chr** | ♂ | ♀ | **Abs**(♂) | **SDr**(♂) | **Chr**(♂) | **Abs**(♀) | **SDr**(♀) | **Chr**(♀) |
| **April** | 594 | 416 | 108 | 70 | 538 | 56 | 366 | 106 | 66 | 50 | 2 | 4 |
| **May** | 992 | 784 | 137 | 71 | 881 | 111 | 689 | 130 | 62 | 95 | 7 | 9 |
| **June** | 1073 | 853 | 149 | 71 | 944 | 129 | 750 | 134 | 60 | 103 | 15 | 11 |
| **July** | 1013 | 796 | 127 | 90 | 885 | 128 | 682 | 118 | 85 | 114 | 9 | 5 |
| **August** | 787 | 606 | 125 | 56 | 691 | 96 | 532 | 116 | 43 | 74 | 9 | 13 |


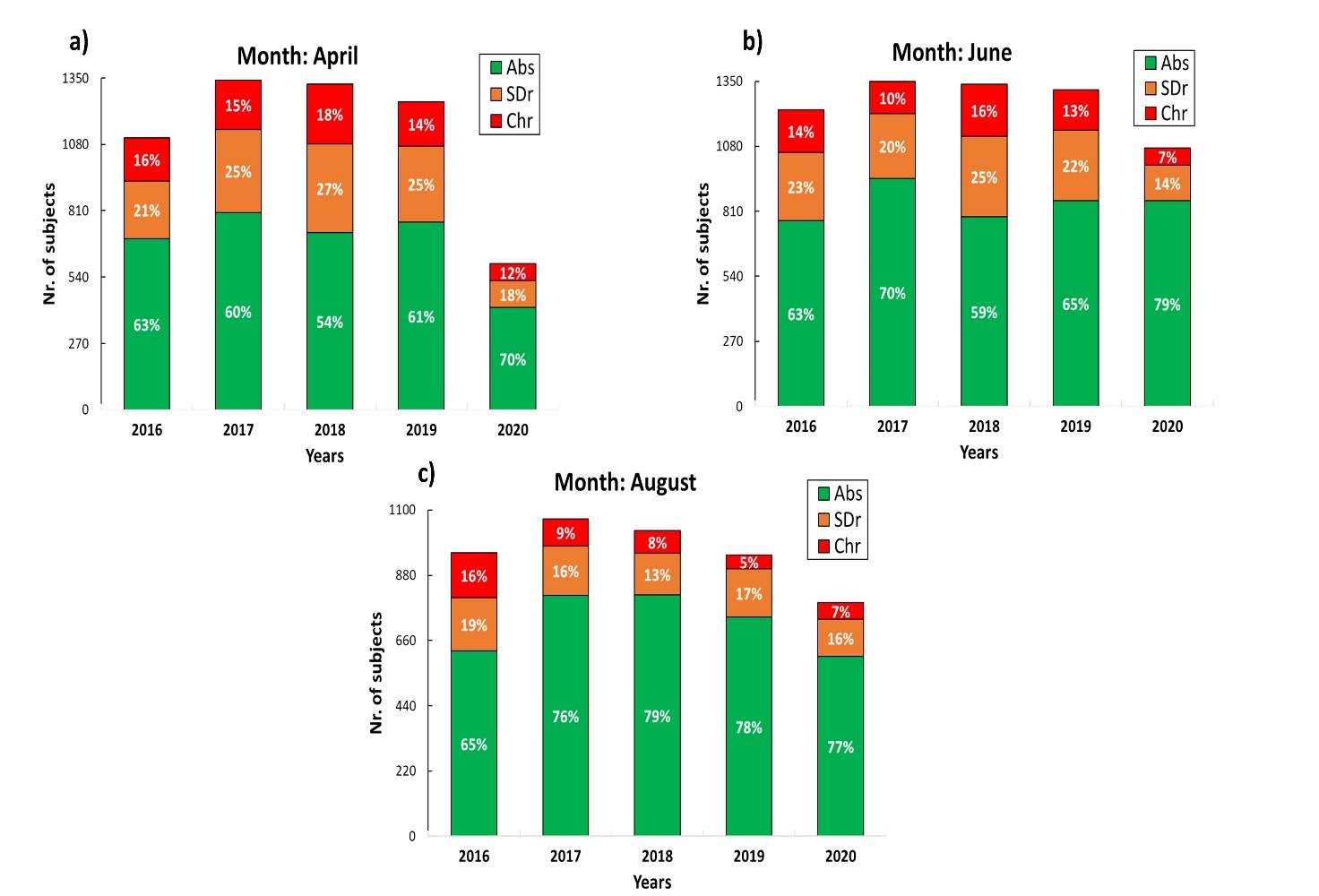


**Figure 1S.** Stacked barplots showing the number and the relative percentages of *Abs* (green), *SDr* (orange), and *Chr* (red) individuals in April (a.), June (b.) and August (c.) 2016-2020.

*The following pie charts (Figure 2S a-k) show the percentage frequencies of the three types of visitors (DRL: driver license reinstatement, TAA: tracked for alcohol abuse, WT: workplace testing) for the year 2020, 2016-2019, and the months' comparisons over time.*


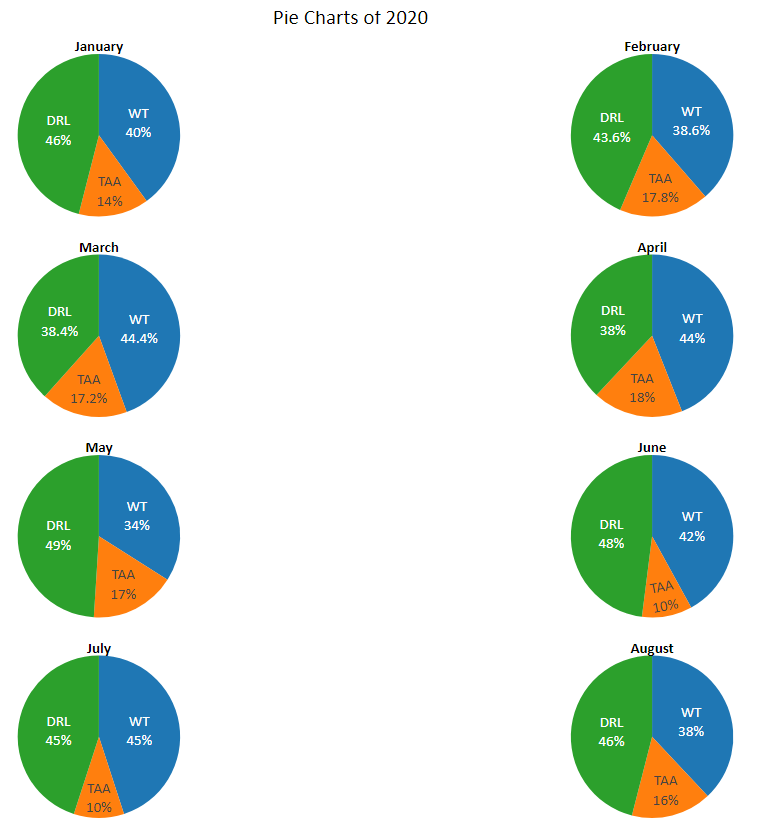


**Figure 2S a.** Pie charts with the percentage frequencies of the three types of visitors (DRL: driver license reinstatement - in green, TAA: tracked for alcohol abuse – in orange, WT: workplace testing – in blue) of the year 2020.


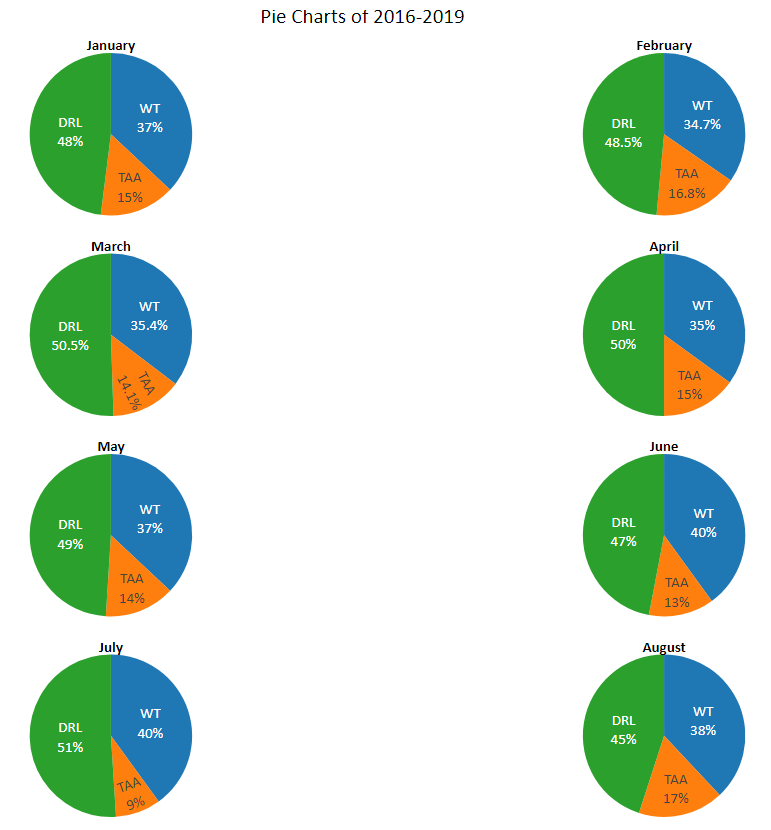


**Figure 2S b.** Pie charts with the percentage frequencies of the three types of visitors (DRL: driver license reinstatement - in green, TAA: tracked for alcohol abuse – in orange, WT: workplace testing – in blue) of the years 2016-2019.


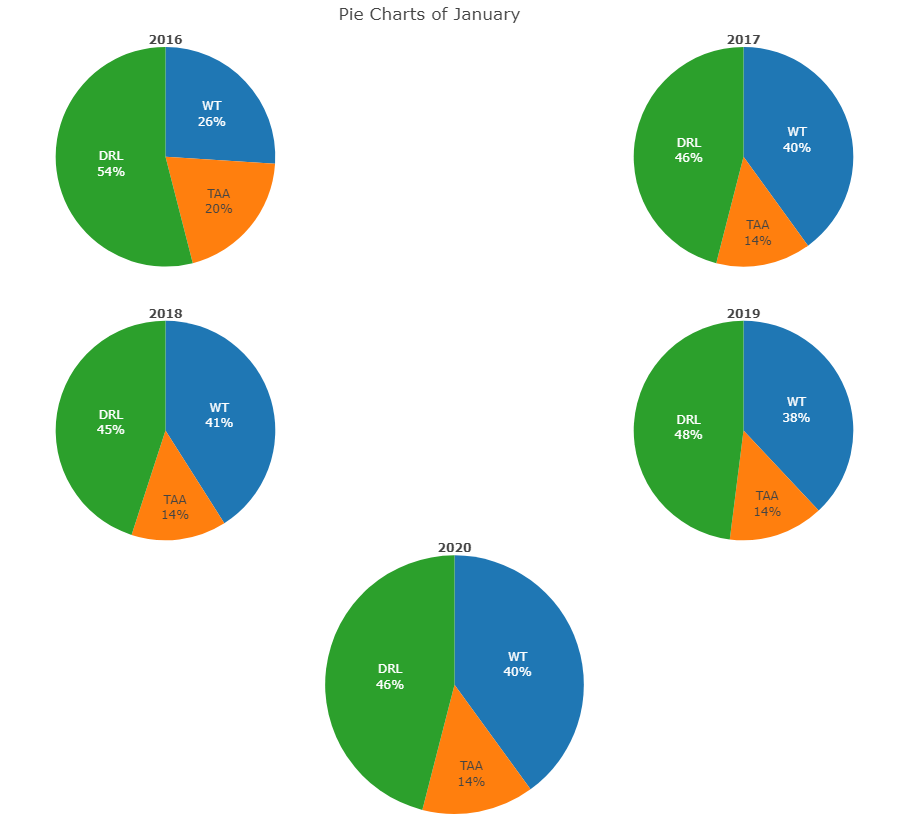


**Figure 2S c.** Pie charts with the percentage frequencies of the three types of visitors (DRL: driver license reinstatement - in green, TAA: tracked for alcohol abuse – in orange, WT: workplace testing – in blue) of January 2020.


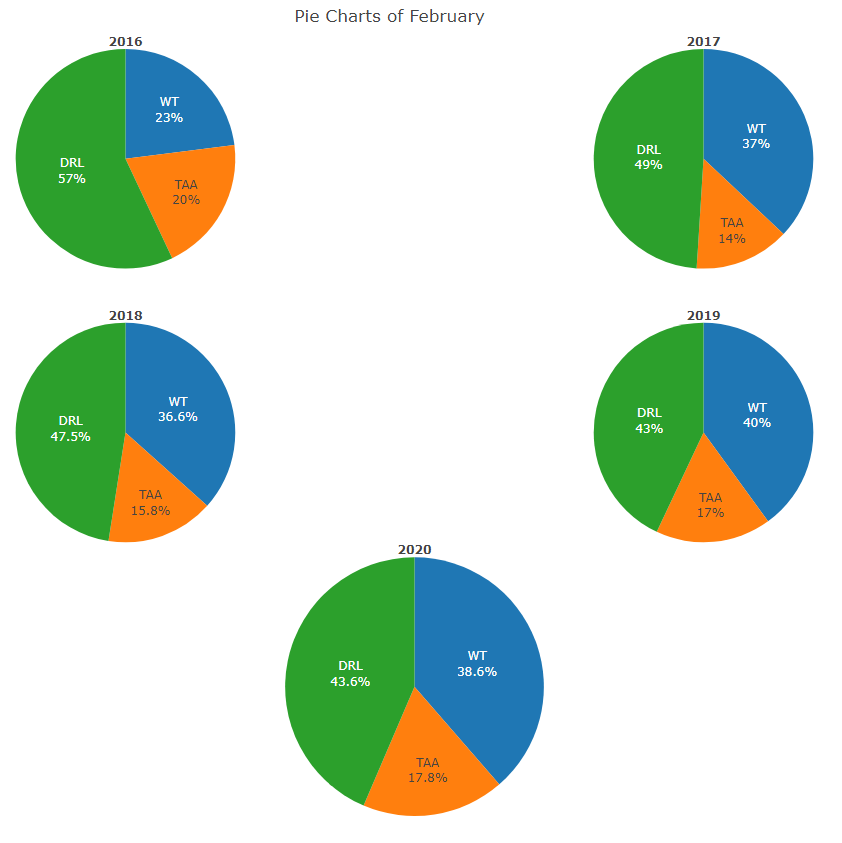


**Figure 2S d.** Pie charts with the percentage frequencies of the three types of visitors (DRL: driver license reinstatement - in green, TAA: tracked for alcohol abuse – in orange, WT: workplace testing – in blue) of February 2020.


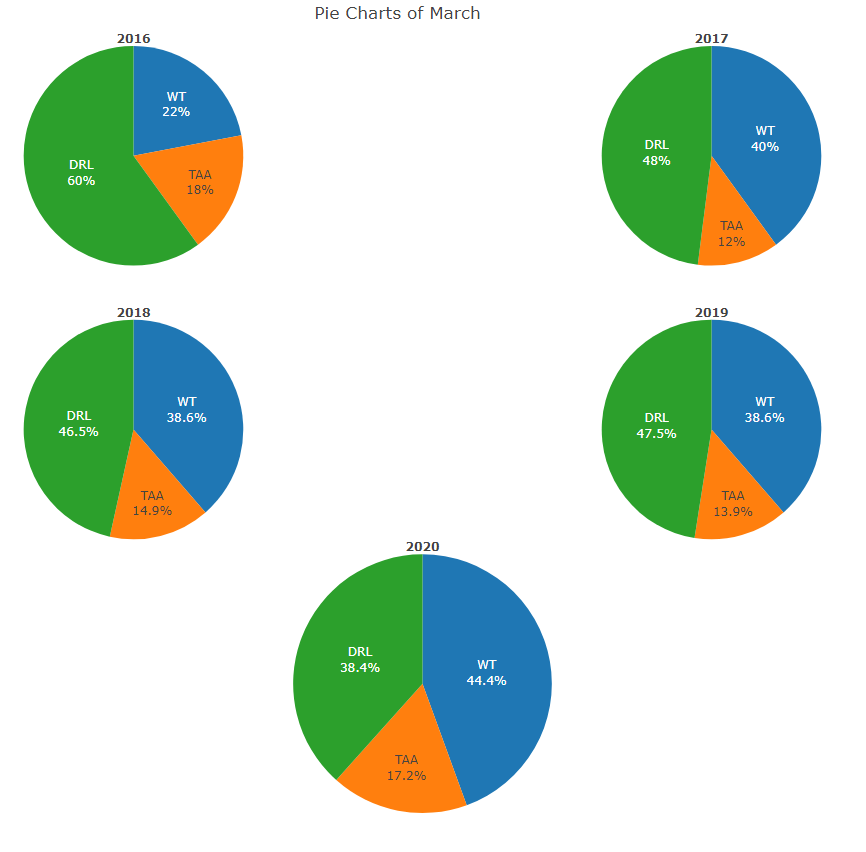


**Figure 2S e.** Pie charts with the percentage frequencies of the three types of visitors (DRL: driver license reinstatement - in green, TAA: tracked for alcohol abuse – in orange, WT: workplace testing – in blue) of March 2020.


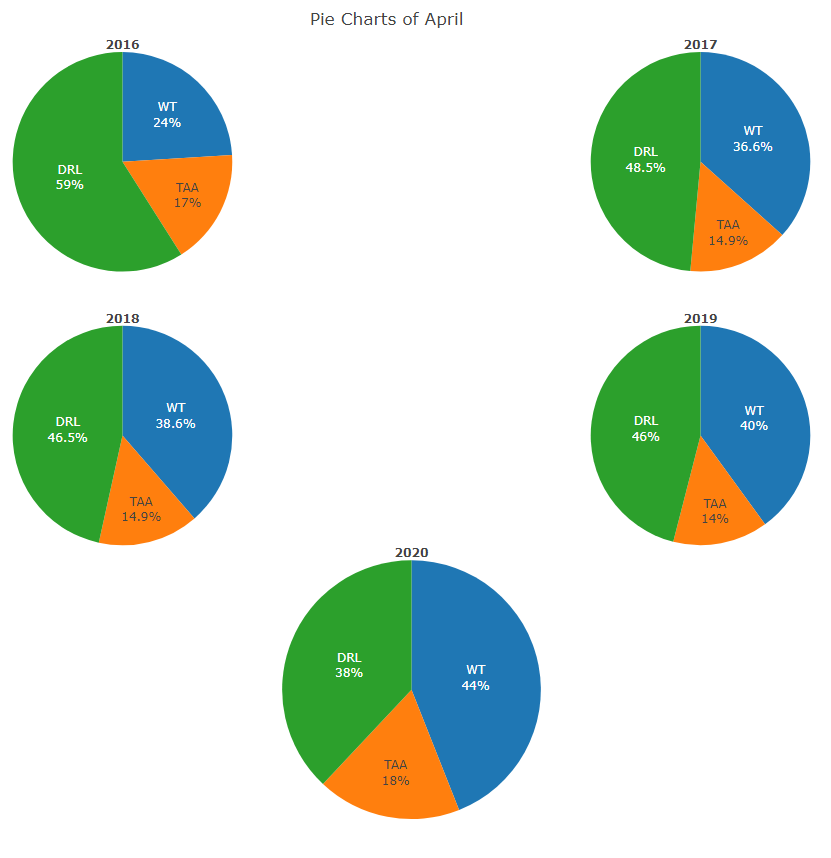


**Figure 2S f.** Pie charts with the percentage frequencies of the three types of visitors (DRL: driver license reinstatement - in green, TAA: tracked for alcohol abuse – in orange, WT: workplace testing – in blue) of April 2020.


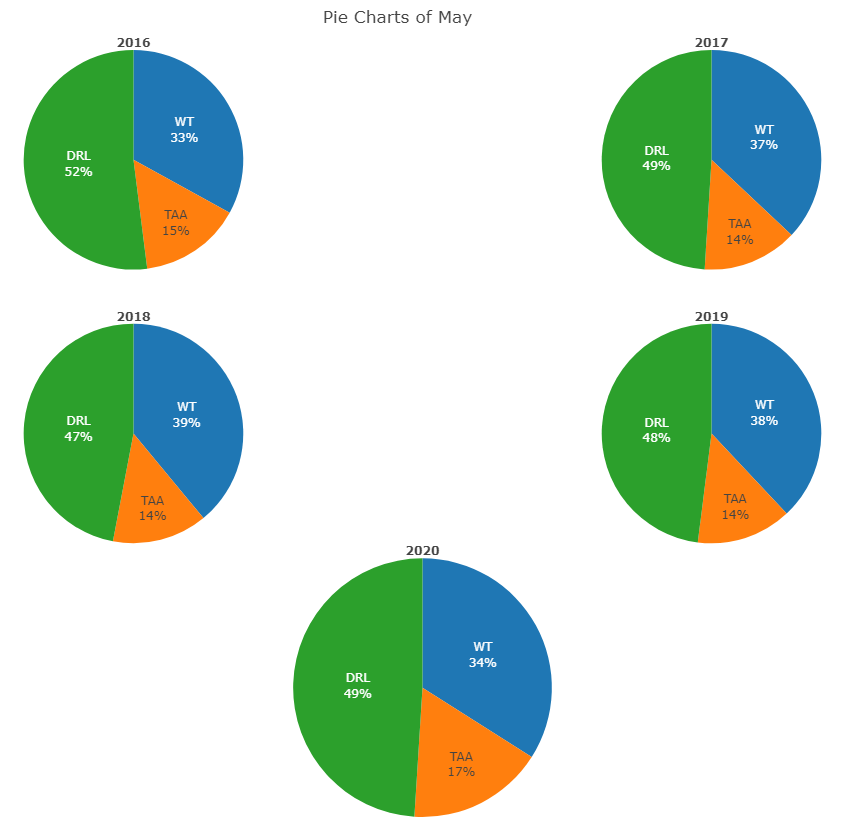


**Figure 2S g.** Pie charts with the percentage frequencies of the three types of visitors (DRL: driver license reinstatement - in green, TAA: tracked for alcohol abuse – in orange, WT: workplace testing – in blue) of May 2020.


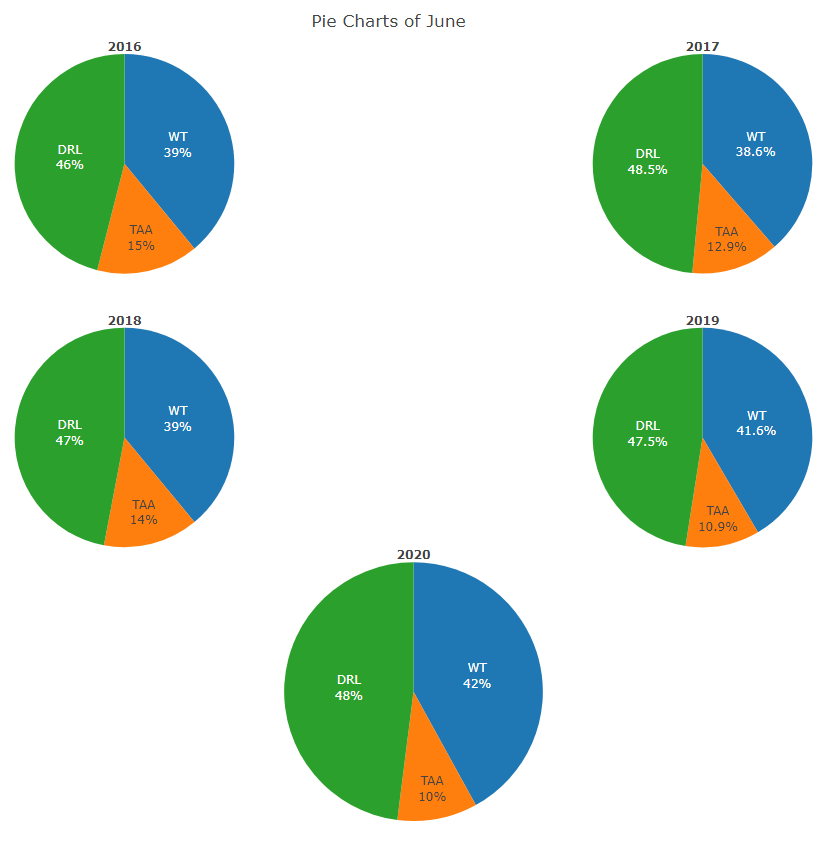


**Figure 2S h.** Pie charts with the percentage frequencies of the three types of visitors (DRL: driver license reinstatement - in green, TAA: tracked for alcohol abuse – in orange, WT: workplace testing – in blue) of June 2020.


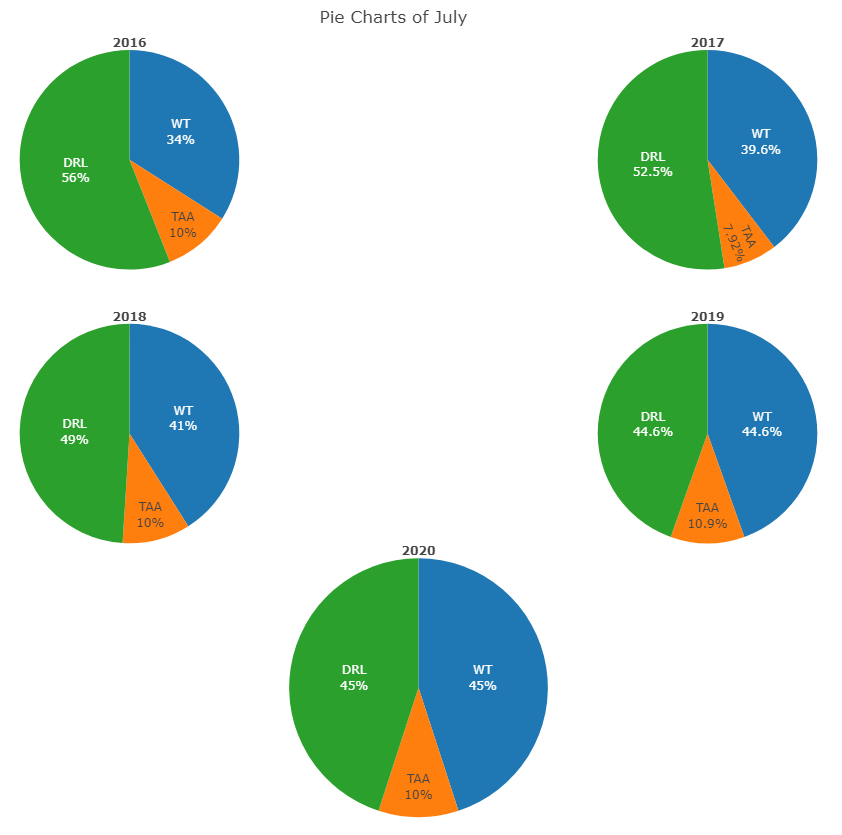


**Figure 2S i.** Pie charts with the percentage frequencies of the three types of visitors (DRL: driver license reinstatement - in green, TAA: tracked for alcohol abuse – in orange, WT: workplace testing – in blue) of July 2020.


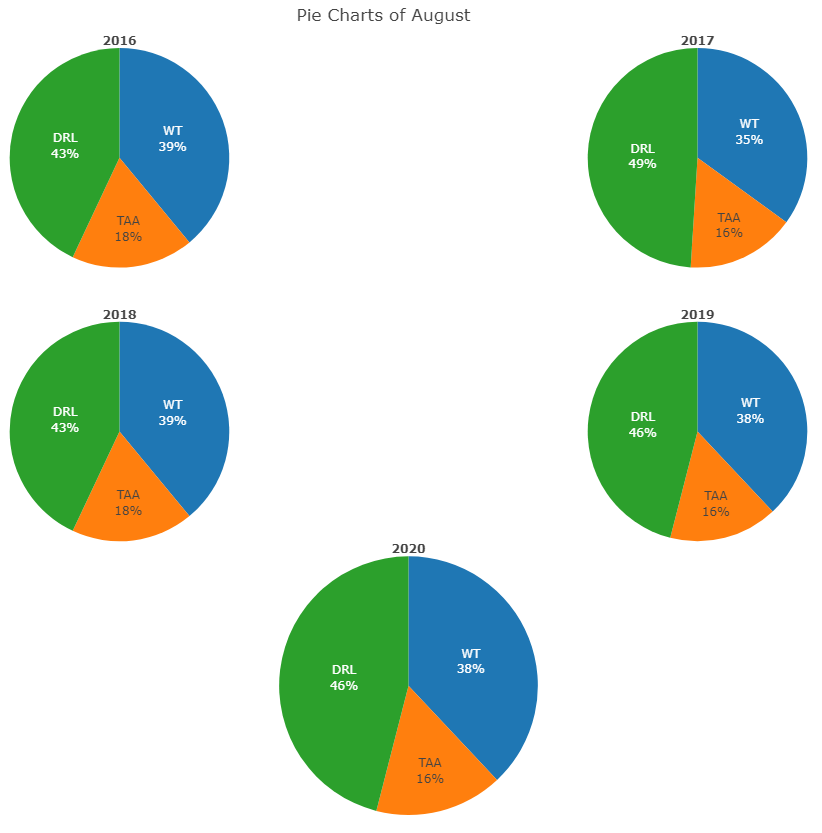


**Figure 2S j.** Pie charts with the percentage frequencies of the three types of visitors (DRL: driver license reinstatement - in green, TAA: tracked for alcohol abuse – in orange, WT: workplace testing – in blue) of August 2020.

The results are variable but relatively consistent over the months. TAA percentages diminish in June and July (together with complementary increases of DRL and WT), while DRL percentages rise in May and August.

No sampling bias effect seemed to occur according to the data, but chi-squared tests were performed for each month (over the tested years 2016-2019 vs. 2020) to dispel any doubt.

The null hypothesis states that the sampled probabilities are the same as the population probabilities and the obtained results indicate a low chance that the null hypothesis is correct. The obtained p-values (reported in the following Table 2S) indicate that we should reject the null hypothesis for several months. However, the observed percentages did not change dramatically, so we believe that there is no bias in interpreting our results. Furthermore, the p-value raises when evaluating 2019 and 2020 only.

**Table 2S.** p-values of the chi-squared tests performed for each month over the tested years 2016-2019 vs. 2020, and 2019 vs. 2020.

| **2016-2019 vs. 2020** | |
| --- | --- |
| **Months** | **p-values** |
| January | 1.30e-01 |
| February | 1.10e-03 |
| March | 2.00e-10 |
| April | 4.30e-08 |
| May | 1.36e-02 |
| June | 1.79e-02 |
| July | 2.84e-03 |
| August | 8.57e-01 |

| **2019 vs. 2020** | |
| --- | --- |
| **Months** | **p-values** |
| January | 2.37e-01 |
| February | 7.46e-01 |
| March | 1.17e-06 |
| April | 2.23e-04 |
| May | 1.36e-03 |
| June | 7.92e-01 |
| July | 7.92e-01 |
| August | 9.60e-01 |


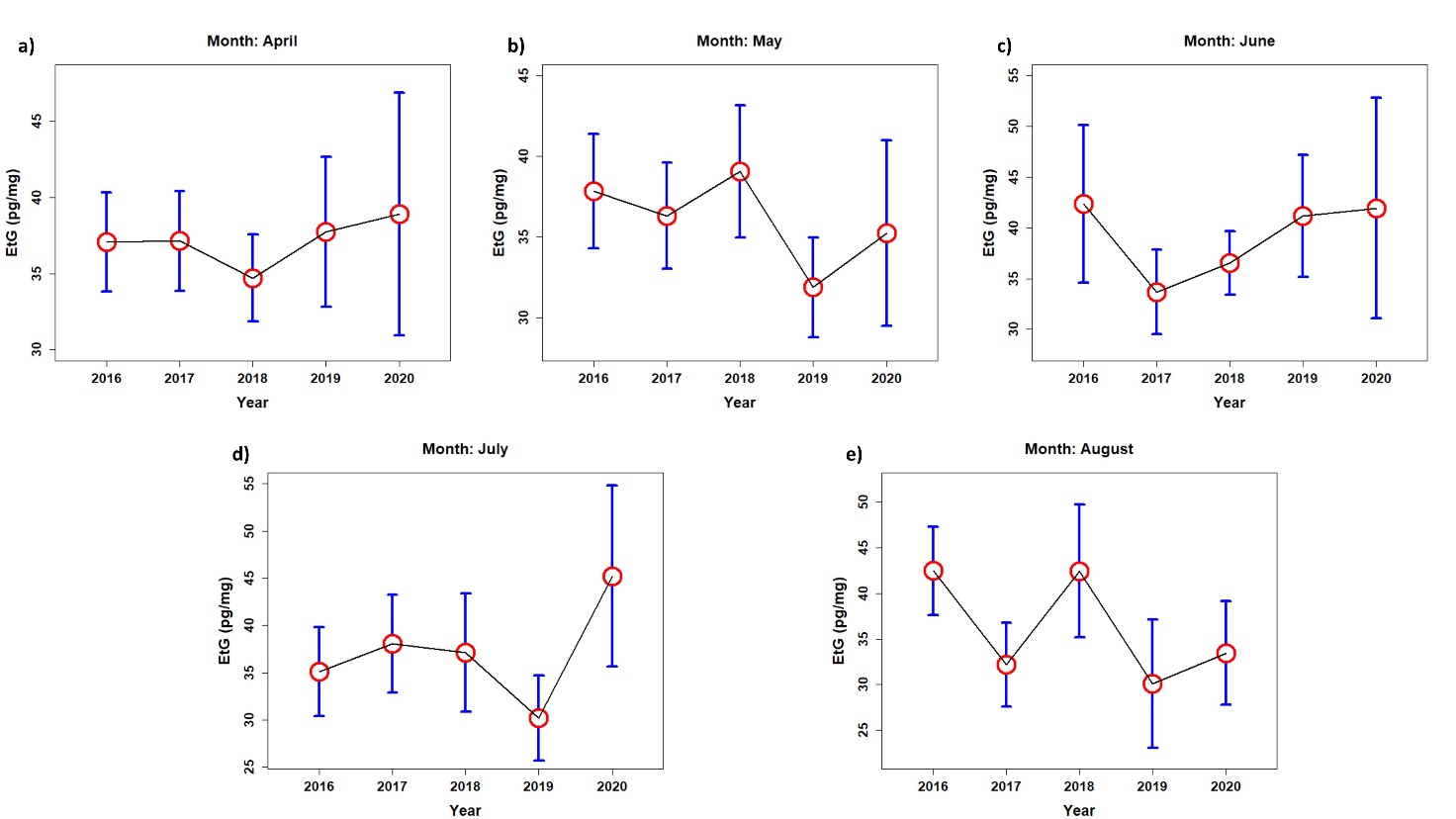


**Figure 3S.** Mean Etg values (red circles) and 95% confidence intervals (blue bars) for all the analyzed hair samples relative to the months of (a.) April, (b.) May, (c.) June, (d.) July, and (e.) August 2016-2020 for the category of chronic/excessive drinkers.

| Table 3S. Percent differences of the EtG mean values of the samples involved in the study divided by years (2016-2020) and months (April-June). *SDr* stands for social/moderate drinkers and *Chr* indicates the chronic/excessive drinkers. Symbols ♂ and ♀ represent the male and female consumers, respectively. | | | | | | | | | |
| --- | --- | --- | --- | --- | --- | --- | --- | --- | --- |
| Overall | | | | | | | | | |
| Year | **Total** | **SDr** | **Chr** | ♂ | ♀ | **SDr**(♂) | **Chr**(♂) | **SDr**(♀) | **Chr**(♀) |
| 2016-2019 | 12.6 | 17.5 | 71.0 | 13.1 | 7.5 | 17.5 | 70.4 | 16.9 | 78.8 |
| 2020 | 9.0 | 17.2 | 78.4 | 9.0 | 9.3 | 17.0 | 76.8 | 19.4 | 104.0 |
| Δ | -3.5 | -0.3 | 7.3 | -4.1 | 1.8 | -0.4 | 6.5 | 2.5 | 25.2 |
| Δ (%) | -28% | -2% | **+10%** | -31% | **+24%** | -3% | **+9%** | **+15%** | **+32%** |

|  | | | | | | | | | |
| --- | --- | --- | --- | --- | --- | --- | --- | --- | --- |
| **April** | | | | | | | | | |
| **Month** | **Total** | **SDr** | **Chr** | ♂ | ♀ | **SDr**(♂) | **Chr**(♂) | **SDr**(♀) | **Chr**(♀) |
| **2016-2019** | 15 | 18 | 66 | 15 | 7 | 18 | 66 | 18 | 70 |
| **2020** | 11.7 | 17.8 | 71.4 | 11.7 | 7.2 | 17.8 | 67.1 | 20.5 | 142.0 |
| **Δ** | -3.1 | -0.3 | 5.9 | -3.7 | 0.1 | -0.4 | 1.6 | 2.5 | 71.9 |
| **Δ (%)** | -21% | -2% | **+9%** | -24% | **+1%** | -2% | **+2%** | **+14%** | **+103%** |
|  |  |  |  |  |  |  |  |  |  |
| **May** | | | | | | | | | |
| **Month** | **Total** | **SDr** | **Chr** | ♂ | ♀ | **SDr**(♂) | **Chr**(♂) | **SDr**(♀) | **Chr**(♀) |
| **2016-2019** | 14 | 18 | 70 | 15 | 5 | 18 | 69 | 17 | 95 |
| **2020** | 7.4 | 17.0 | 70.4 | 7.4 | 7.8 | 16.9 | 69.1 | 19.3 | 79.0 |
| **Δ** | -7.1 | -0.5 | 0.5 | -7.6 | 2.3 | -0.7 | 0.5 | 2.2 | -16.4 |
| **Δ (%)** | -49% | -3% | **+1%** | -51% | **+42%** | -4% | **+1%** | **+13%** | -17% |
|  |  |  |  |  |  |  |  |  |  |
| **June** | | | | | | | | | |
| **Month** | **Total** | **SDr** | **Chr** | ♂ | ♀ | **SDr**(♂) | **Chr**(♂) | **SDr**(♀) | **Chr**(♀) |
| **2016-2019** | 14 | 18 | 74 | 15 | 8 | 18 | 74 | 16 | 63 |
| **2020** | 8.6 | 17.0 | 94.3 | 8.7 | 9.9 | 17.1 | 98.1 | 16.1 | 73.6 |
| **Δ** | -5.2 | -0.6 | 20.3 | -5.9 | 1.8 | -0.6 | 23.8 | -0.2 | 10.2 |
| **Δ (%)** | -38% | -3% | **+27%** | -40% | **+22%** | -3% | **+32%** | -1% | **+16%** |
|  |  |  |  |  |  |  |  |  |  |
| **2019** | | | | | | | | | |
| **Month** | **Total** | **SDr** | **Chr** | ♂ | ♀ | **SDr**(♂) | **Chr**(♂) | **SDr**(♀) | **Chr**(♀) |
| **2016-2019** | 10 | 17 | 71 | 11 | 8 | 17 | 72 | 16 | 49 |
| **2020** | 9.7 | 17.3 | 84.6 | 10.1 | 9.0 | 17.1 | 80.9 | 20.7 | 147.0 |
| **Δ** | -0.4 | 0.2 | 13.4 | -0.6 | 0.8 | -0.1 | 8.5 | 4.6 | 97.9 |
| **Δ (%)** | -4% | **+1%** | **+19%** | -6% | **+9%** | 0% | **+12%** | **+29%** | **+200%** |
|  |  |  |  |  |  |  |  |  |  |
| **2020** | | | | | | | | | |
| **Month** | **Total** | **SDr** | **Chr** | ♂ | ♀ | **SDr**(♂) | **Chr**(♂) | **SDr**(♀) | **Chr**(♀) |
| **2016-2019** | 10 | 17 | 75 | 10 | 8 | 17 | 71 | 17 | 116 |
| **2020** | 7.7 | 16.6 | 71.1 | 7.0 | 12.6 | 16.3 | 68.9 | 20.6 | 78.5 |
| **Δ** | -1.8 | -0.3 | -3.5 | -2.6 | 4.1 | -0.5 | -2.1 | 3.4 | -37.7 |
| **Δ (%)** | -19% | -2% | -5% | -27% | **+48%** | -3% | -3% | **+20%** | -32% |
