# Supplementary figures and images for: The Impact of COVID-19 Pandemic and Lockdown on Alcohol Consumption: A Perspective From Hair Analysis
Source: Front Psychiatry. 2021 Apr 6;12:632519. doi: 10.3389/fpsyt.2021.632519 (PMC8055823; doi:10.3389/fpsyt.2021.632519)

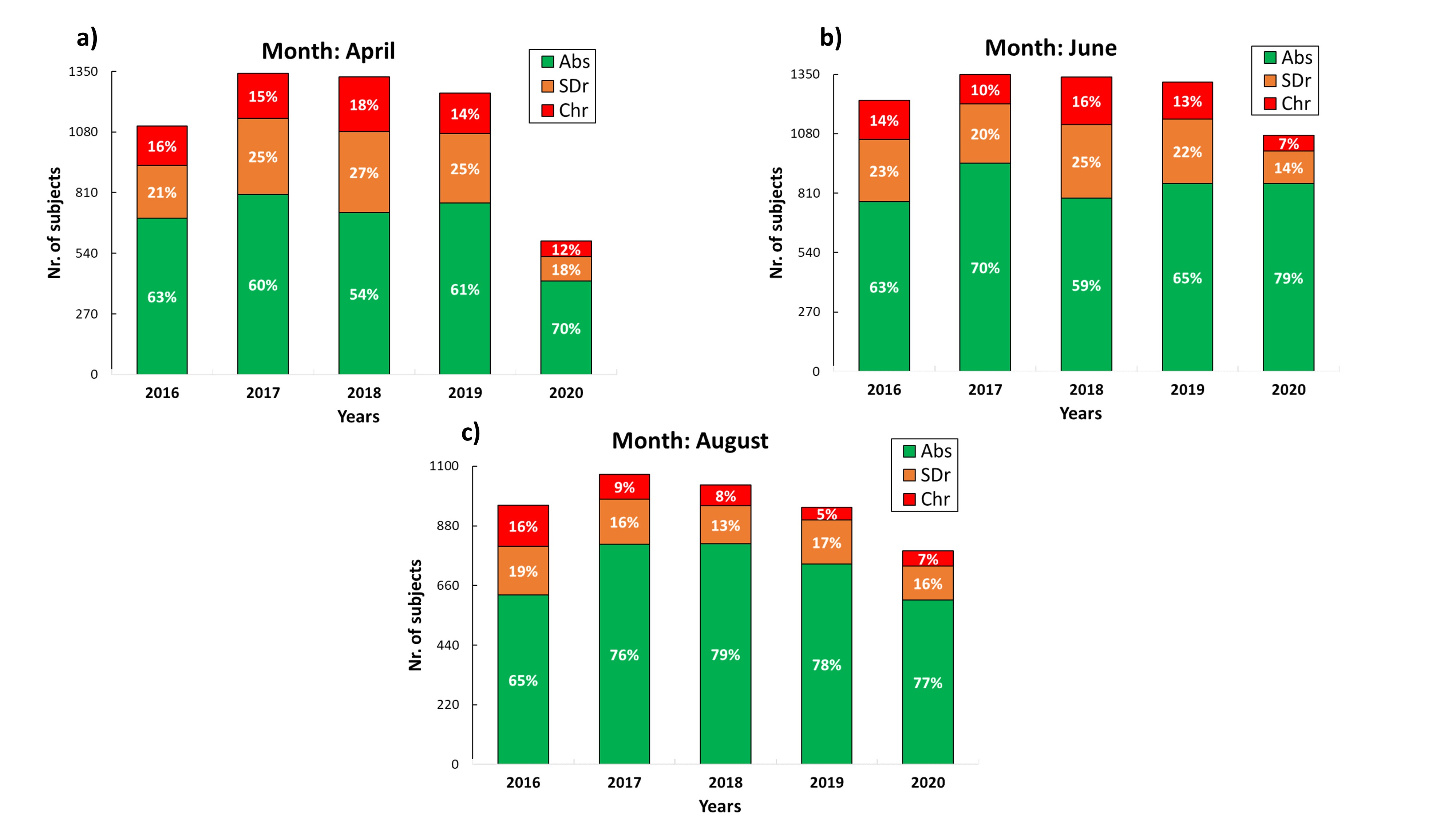

Supplement: Supplementary file 2 [file Image_1.JPEG]

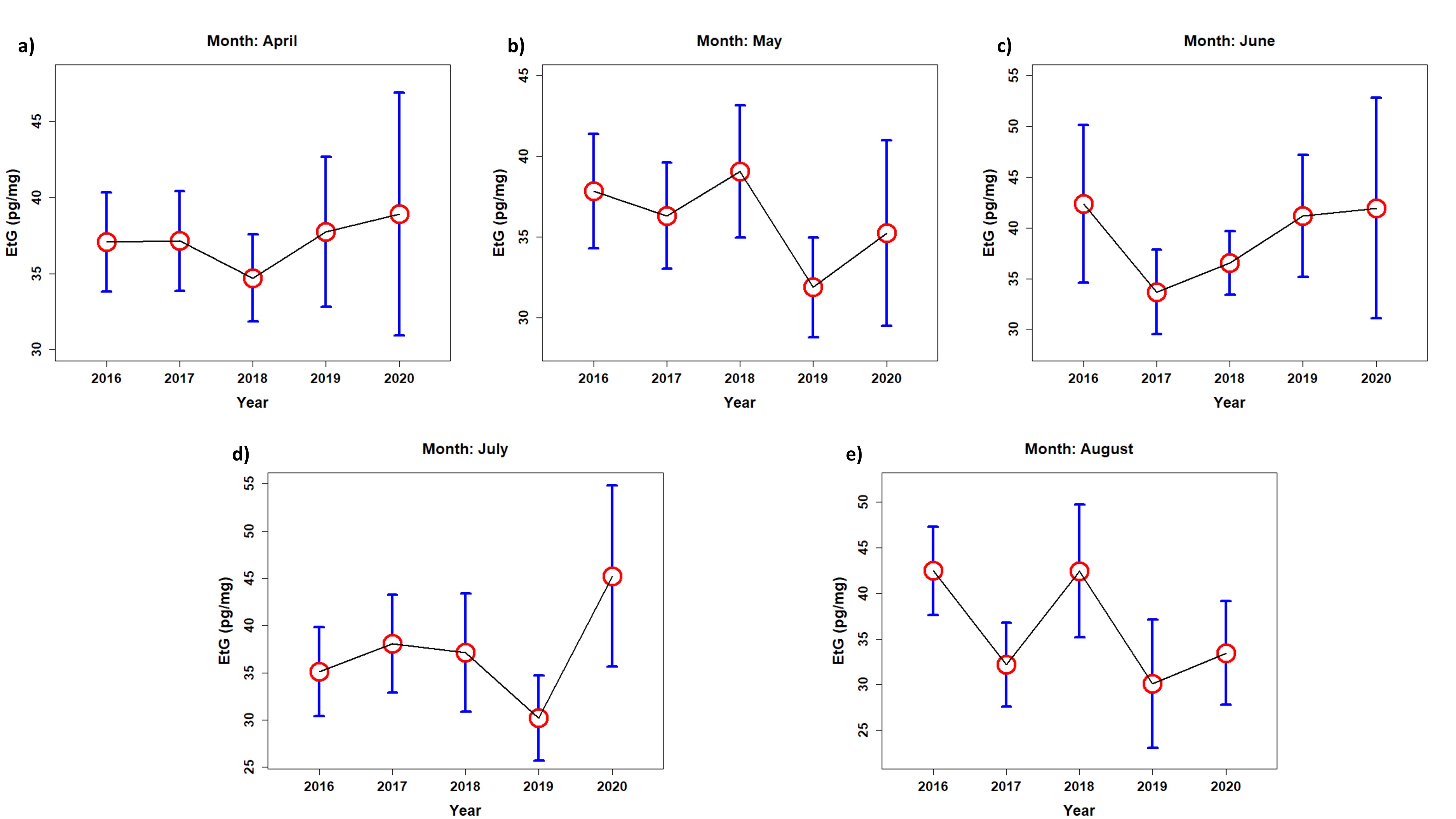

Supplement: Supplementary file 3 [file Image_2.JPEG]
